# Establishment of stably expandable induced myogenic stem cells by four transcription factors

**DOI:** 10.1038/s41419-018-1114-8

**Published:** 2018-10-25

**Authors:** Eun-Joo Lee, Minhyung Kim, Yong Deuk Kim, Myung-Jin Chung, Ahmed Elfadl, H. M. Arif Ulah, Dongsu Park, Sunray Lee, Hyun-Sook Park, Tae-Hwan Kim, Daehee Hwang, Kyu-Shik Jeong

**Affiliations:** 1 Department of Veterinary Pathology, College of Veterinary Medicine, Kyungpook National University, Daegu, 41566 Republic of Korea; 2 School of Interdisciplinary Bioscience and Bioengineering, Pohang University of Science and Technology, Pohang, 37673 Republic of Korea; 3 Stem Cell Therapeutic Research Institute, Kyungpook National University, Daegu, 41566 Republic of Korea; 4 Department of Molecular Human Genetics, Baylor College of Medicine, One Baylor Plaza, Houston, TX 77030 USA; 5 Center for Skeletal Biology, Baylor College of Medicine, One Baylor Plaza, Houston, TX 77030 USA; 6 Department of Pathology and Immunology, Baylor College of Medicine, One Baylor Plaza, Houston, TX 77030 USA; 7 Cell Engineering for Origin Research Center 45-13, Ujeongguk-ro, Jongno-gu, Seoul 03150 Republic of Korea; 8 Center for Plant Aging Research, Institute for Basic Science, Daegu Gyeongbuk Institute of Science and Technology (DGIST), Daegu, 42988 Republic of Korea; 9 Department of New Biology, DGIST, Daegu, 42988 Republic of Korea

## Abstract

Life-long regeneration of healthy muscle by cell transplantation is an ideal therapy for patients with degenerative muscle diseases. Yet, obtaining muscle stem cells from patients is very limited due to their exhaustion in disease condition. Thus, development of a method to obtain healthy myogenic stem cells is required. Here, we showed that the four transcription factors, Six1, Eya1, Esrrb, and Pax3, converts fibroblasts into induced myogenic stem cells (iMSCs). The iMSCs showed effective differentiation into multinucleated myotubes and also higher proliferation capacity than muscle derived stem cells both in vitro and in vivo. The iMSCs do not lose their proliferation capacity though the passaging number is increased. We further isolated CD106-negative and α7-integrin-positive iMSCs (sort-iMSCs) showing higher myogenic differentiation capacity than iMSCs. Moreover, genome-wide transcriptomic analysis of iMSCs and sort-iMSCs, followed by network analysis, revealed the genes and signaling pathways associated with enhanced proliferation and differentiation capacity of iMSCs and sort-iMSCs, respectively. The stably expandable iMSCs provide a new source for drug screening and muscle regenerative therapy for muscle wasting disease.

## Introduction

Dysfunction of muscle stem cells causes muscle diseases. Although there is much progress in understanding the genetic defects in degenerative muscle diseases, the diseases remain incurable. Therefore, the transplantation of stem cells to damaged muscle tissue has been considered as an ideal therapeutic strategy.

Autologous stem cell transplantation is by far the most common stem cell transplantation. In degenerative muscle diseases, however, application of muscle derived stem cells (MDSC) from patients has been technically challenging. It is very hard to isolate myoblasts or satellite cells from muscular dystrophy or sarcopenia patients, as these patients show difficulties in regenerating muscle and have less muscle than normal individuals. Additionally, muscle laceration for sampling is very invasive, especially in case of patients with muscular dystrophy or sarcopenia. Further, MDSCs, as multipotent, have the differentiation capacity that is not confined only to myogenesis, but also to adipogenesis, chondrogenesis and osteogenesis^1^. An animal study also showed that satellite cells under aging become fibrogenic rather than myogenic via the activation of Wnt signaling^2^. Thus, a new source for myogenic stem cells that can be used in stem cell therapy is necessary.

It has been revealed that terminally differentiated somatic cells can be directly converted into totally different cell types by forcing ectopic expression of specific transcription factors (TFs), referred to as direct conversion. Various cell types, including neurons^3,4^, hepatocytes^5,6^, cardiomyocytes^7–9^, and blood progenitor cells^10^ were induced from completely different somatic cells using tissue-specific TFs.

The concept that ectopic expression of cell type-specific genes alters cell properties was first reported by Davis et al.^11^ in 1987. They revealed that transfected MyoD cDNA converts C3H10T1/2 embryonic fibroblasts into myoblasts. MyoD, a well-known muscle specific TF, converts primary cells including dermal fibroblasts, chondroblasts, smooth muscle, and retinal pigmented epithelial cells into myoblasts and myotubes^12^. Also, Liu et al. suggested that ectopic expression of MyoD and Cx43 make fibroblasts differentiate into muscle fibers^13^. MyoD can induce myogenic differentiation from non-myogenic cells. However, MyoD inhibits proliferation of these transdifferentiated cells and these cells do not have self-renewing and expansion capacities. The proliferation capacity is important for the use of the generated cells because sufficient cells are required for cell transplantation and drug screening. No studies have established stably expandable myogenic stem cells by direct reprogramming, although Naoki et al. induced myogenic progenitor cells with the combination of six transcription factors^14^.

*Sine Oculis* (*Six1*) plays a critical role in muscle development. Loss of *Six1* leads to lethality of fetus. The fetuses of *Six1* mutant mice exhibit impaired primary myogenesis and muscle defects in the diaphragm, forelimb, and hindlimb^15^. Overexpression of *Six1* increases the expression of Pax3^16^. Six1 promotes the proliferation of Pax7 ( + ) cells by upregulating Smad1/5/8^17^. *Eyes absent*1 (Eya1) functions as a cofactor of *Six1* to activate *Six1* target genes including Pax3, MyoD and myogenin^18,19^. *Eya1/Eya2* mutant mice show delayed myogenesis during development^18^. *Estrogen related receptor beta* (*Esrrb*) is a TF that upregulates the self-renewal of trophoblasts and embryonic stem cells^20,21^. *Paired box 3* (*Pax3*) plays a crucial role in the formation of ventro-lateral dermomyotome, which develops into the hypaxial body and limb muscle during the development^22–24^.

In this study, by using a novel combination of these four TFs involved in myogenesis or self-renewal, we have established stably expandable induced myogenic stem cells from fibroblasts and revealed its in vitro and in vivo proliferation and myogenic differentiation capacity.

## Materials and methods

### Lentivirus preparation

The *Six1* open reading frame was subcloned into the pLJM lentiviral vector (Addgene no. 19319), which have a puromycin resistance gene. *Eya1* and *Pax3* were subcloned into the FUW-tet-O plasmid (Addgene no. 20321). A plasmid containing *Esrrb* (Addgene no. 40798) was purchased from Addgene. A total of 5 × 10^6^ of 293 FT cells were plated onto a 100 mm dish. When the 293 FT cells were 90% confluent, they were transfected with 3 µg of lentiviral vector delivered by 36 µL Lipofectamine 2000 (Invitrogen), 5 mL of opti-MEM, and 9 µg of ViraPower lentiviral packaging mixture (Invitrogen). Viral supernatant was harvested 48 h after transfection, centrifuged at 3000 rpm for 15 min at 4 °C, and filtered through a 0.45 µM filter (Millipore). The titration of viruses was greater than 5 × 10^5^ IFU/mL according to measurement with Lenti-X GoStix (Clontech).

### Establishment of iMSCs

To isolate mouse embryonic fibroblasts (MEFs), mouse embryos (C57BL/6J) were isolated from the uterus of pregnant female mice at E13.5 days. Head and red organs were eliminated and remaining whole body parts were cut and minced. The minced tissues were incubated for 10 min at 37 °C with 0.05% of trypsin-EDTA for enzymatic digestion and then the enzyme was neutralized. The pellet was suspended in media and filtered through 0.45 µm filters MEFs were maintained in MEF media (10% fetal bovine serum (FBS), 1% penicillin/streptomycin in high glucose DMEM). The MEFs were maintained until passage 3 and seeded at 5 × 10^4^. The MEFs were incubated with Six1 lentivirus for 48 h. Two days after transduction, the culture media was replaced with 1.5 mL of myogenic growth media (10% FBS, 10% horse serum, 5 ng/mL murine basic fibroblast growth factor (FGF) and 1% penicillin/streptomycin in high glucose DMEM) and the cells were treated with 5 µg/mL puromycin for the next 25 days. The media was changed every 2 days. After finishing puromycin selection, the Six1 transduced cells were seeded at 5 × 10^4^ and transduced with the lentivirus of Esrrb, Eya1, and Pax3. After 48 h of transduction with lentivirus of Esrrb, Eya1, and Pax3, the media containing lentivirus was replaced with myogenic growth media. The cells were maintained for 7 days with doxycycline (2 µg/mL) to turn on the tet-O system. When the cells showed robust proliferation, single-cell sorting was performed using the FACS Aria and single-cell lines were established. Each single-cell line was propagated and after cell growth and RNA purification, PCR was performed for Pax7 and Myf5.

### Real-time reverse transcriptase-polymerase chain reaction (real-time RT-PCR) analysis

RNA was isolated using Trizol following the manufacturer’s protocol. cDNA was synthesized using 0.5 µg of the RNA with reverse transcriptase (Invitrogen). For real-time RT-PCR, 2 × SYBR Green PCR mastermix was used. The primers for Pax7 (QT00147728), Myf5 (QT00199507), MyoD (QT00101983), myogenin (QT00112378), MHC (QT0106850), and GAPDH (QT01658692) were purchased from Qiagen. Rotorgene Q (Qiagen) was used to perform real-time RT-PCR and analyze the results.

### Immunofluorescence (IF)

Cells were grown on 2% gelatin-coated multi-well plates and fixed for 15 min at room temperature in 4% paraformaldehyde. After aspirating fixative and washing with phosphate-buffered saline (PBS), samples were incubated for 10 min at −20 °C in 100% methanol for permeabilization and then rinsed three times with PBS for 5 min each. Samples were then blocked for 1 h at room temperature with 5% BSA. After aspirating the blocking buffer, the samples were incubated with primary antibodies against Pax7 (1:50, Developmental Studies Hybridoma Bank), Myf5 (1:200, Santa Cruz), MyoD (1:100, Santa Cruz), myogenin (1:25, Developmental Studies Hybridoma Bank), and MHC (1:100, Santa Cruz) for overnight at 4 °C. The samples were rinsed three times with PBS for 5 min each. The samples were incubated in alexar fluor 594- or TRITC-conjugated secondary antibodies (1:400) for 1 h at room temperature. For counter staining, the samples were incubated in 4′, 6-diamidino-2-phenylindole (DAPI) for 10 min and mounted with coverslip.

### Immunoblot analysis

Cells were washed with cold PBS and harvested with RIPA buffer containing complete protease inhibitor cocktail (Roche). Protein concentration was measured according to the Bradford method^25^. Next, 50 µg of total protein was incubated at 100 °C for 10 min with electrophoresis sample buffer. The samples were loaded on each well and separated by 6–20% SDS-polyacrylamide gel. Proteins were transferred to a polyvinylidene difluoride (PVDF) membrane. The blot was blocked with 5% skim milk for 1 h at room temperature and incubated overnight at 4 °C with primary antibodies against Six1 (1:1000, Cell Signaling), Eya1 (1:500, Abcam), Pax3 (1:500, R&D Systems), Esrrb (1:1000, R&D Systems), GRB2 (1:1000, Santa Cruz), and GAPDH (1:2000, Cell Signaling). After incubation with secondary antibodies (1:2000, Cell Signaling) conjugated to horseradish peroxidase, signals were detected by Super Signal West Dura Extended Duration Substrate (Pierce). The signals were visualized using an Image Analyzer (UVP) or exposed to medical X-ray film.

### Myogenic differentiation in vitro

To evaluate myogenesis in vitro, cells were seeded onto a 2% gelatin-coated dish and incubated until the cells occupied 90% of the culture dish area. Next, the dishes were washed with PBS and the media was changed to myogenic differentiation media. The myogenic differentiation media contained 2% horse serum and 1% penicillin/streptomycin in low-glucose DMEM. Cells were incubated for 3 days in myogenic differentiation media. The media was changed every day. Differentiated cells were analyzed by real-time RT-PCR and IF.

### Proliferation test in vitro

To evaluate proliferation capacity, 3 × 10^4^ cells were seeded onto a 2% gelatin-coated dish. The trypsinized cells were stained with trypan blue. The number of cells was counted every 24 h using a hemocytometer and trypan blue. This experiment was repeated three times.

### Animals

Mice were maintained in a room at 22 ± 3 °C, relative humidity 50 ± 10%, and 12 h light-dark cycle and were given food and water ad libitum. Animal experiments were performed in accordance with the NIH guidelines for the care and use of laboratory animals and approved by institutional animal care and use committee of Kyungpook National University (Approval no. 2014–0167).

### Myogenic differentiation in vivo

For in vivo myogenic differentiation analysis, 4-weeks-old male mdx mice were used in vivo. We injected notexin into the middle of tibialis anterior (TA) muscle at a dose of 50 µg to injure the muscle one day before cell transplantation. The iMSCs were prepared and 30,000 cells were suspended in 20 µL of PBS. The cells were transplanted using an insulin syringe. The tibialis anterior muscle was harvested from mice 4 weeks after cell transplantation. To detect dystrophin, staining was performed as previously described^26^. Briefly, frozen sections were blocked overnight at 4 °C with 10% horse serum in PBS, and then probed overnight at 4 °C with rabbit anti-dystrophin antibody (Abcam, 1:100). TRITC-conjugated anti-rabbit IgG was used as the secondary antibody (Invitrogen, 1:400). Images were obtained by confocal fluorescence microscopy.

### Intravital imaging of iMSCs

pWPXL-based lentiviral vector expressing tdTomato (here after Tomato) was kindly provided by Xiang Zhang from Baylor College Medical School. To generate iRFP670-expressing lentivirus, the EGFP gene in pWPXL lentiviral vector (Addgene) was removed by BamHI/EcorRI digestion and full-length iRFP670 from the piRFP670 plasmid (Addgene) obtained by BamHI/MfeI digestion was cloned into *Bam*HI/*Eco*rRI sites of pWPXL. pWPXL-based lentivirus was produced using HEK 293T cells. To visualize transplanted iMSCs in muscle regeneration, in vitro cultured MDSCs and iMSCs were transduced with iRFP670-expressing lentivirus or tdTomato-expressing lentivirus respectively. Tomato^+^ iMSCs and iRFP670^+^ MDSCs were sorted by FACS and expanded for transplantation analysis. Muscle injury and stem cell transplantation was performed as described previously^27,28^ with minor modifications. Briefly, right TA muscle was injured with 50 μl of barium chloride (1.2%) with 10–20 multiple needle punctures for stem cell transplantation. Twenty-four hours after injury, in vitro cultured Tomato^+^ iMSCs and iRFP670^+^ MDSCs and iMSCs (1 × 10^5^/50 µL) were transplanted into injured muscle. To sequentially image the muscle after cell engraftment in vivo, mice were anesthetized and prepared for customized multiphoton/confocal hybrid microscope system (Leica TCS SP8 MP platform) specifically designed for live animal imaging as described previously^29^. After the mouse was mounted on a motorized stage, the exposed and injured TA muscle was scanned for second harmonic generation (SHG by femto-second titanium:sapphire laser pulses tuned to 880 nm: Chameleon Vision Laser, Coherent) from muscle fibers. Tomato- (554 nm excitation, 581 nm detection) or iRFP670-expressing cells (630 nm excitation, 670 nm detection) in combination with myofibers (440 nm s harmonic signals) were simultaneously imaged by intravital microscopy. To identify the imaging location of the TA muscle and to repeat the imaging sequentially, the xyz coordinates of imaging position from the lateral tibia head (a landmark position) were recorded.

### Tumor formation assay

Six-week-old male nude mice (balb/c nu/nu mice) were used in this assay. First, 5 × 10^6^ iMSCs and iPSCs were suspended in PBS. The cells were injected subcutaneously into the dorsal part of the nude mice. After 3 weeks, mice were sacrificed and the tumor tissues from the injected site were isolated for histopathological analysis. Tumor tissues were fixed in 10% neutral-buffered formalin, processed routinely, and embedded in paraffin wax. The sections were cut to 4-µm thickness and then deparaffinized in toluene and rehydrated in a graded alcohol series. The sections were stained with hematoxylin and eosin (H&E).

### mRNA microarray experiments

Total RNA was isolated from MEFs, MDSCs, iMSCs, and sort-fiMSC cells using the RNeasy mini kit (Qiagen). RNAs were obtained from two independent biological replicates. RNA integrity was assessed using the Agilent 2100 Bioanalyzer, and RNA integrity numbers for all samples were above 8.0. RNA was reverse-transcribed, amplified, and hybridized to the Agilent SurePrint G3 mouse GE 8 × 60 K microarray, including 62,976 probes corresponding to 23,853 genes, according to Agilent’s protocols. Probe intensities were obtained using the Agilent G2565BA microarray scanner and then normalized using the quantile normalization method^30^. The microarray data were deposited to the gene expression omnibus (GEO) database (Accession ID: GSE94506).

### Statistical analysis of gene expression data

We identified differentially expressed genes (DEGs) from the three comparisons (MDSC vs. MEF, iMSC vs. MEF, and sort-iMSC vs. MEF) using a previously reported statistical test^31^. Briefly, a *T*-value was computed for each gene. An empirical distribution of the null hypothesis (i.e., a gene is not differentially expressed) was estimated by calculating *T*-values for the genes after randomly permuting the samples and by applying the Gaussian density estimation method to the *T*-values obtained from the random permutations. For each gene, the adjusted *P*-value was computed by performing the two-tailed *T*-test for its *T*-value using the empirical null distribution. DEGs were selected as the genes with (1) adjusted *P*-values ≤ 0.05 and 2) absolute log_2_-fold-changes ≥ 1. Finally, the enrichment analysis of gene ontology biological processes (GOBPs) was performed for a list of genes using DAVID software^32^ and the GOBPs represented by the genes were identified as those with the enriched *P*-values < 0.05 (EASE test in DAVID).

### Network analysis

To build a network model for muscle differentiation defined by iMSCs and sort-iMSC cells, we first selected the cell cycle- and differentiation-related GOBPs represented by the DEGs. We then obtained the DEGs involved in these processes and identified KEGG pathways represented by such DEGs: Calcium, Jak-STAT, MAPK, Notch, TGF-ß, VEGF, and Wnt signaling pathways, as well as the pathway for the regulation of the actin cytoskeleton. Next, we built a network model describing the interactions among the DEGs involved in these pathways using interaction data in the KEGG pathways and also protein–protein interactions (PPIs) collected from the following PPI databases: the Biological General Repository for Interaction Datasets (BioGRID)^33^, the Database of Interacting Proteins (DIP)^34^, High confidence protein–protein interactions (HitPredict)^35^, the IntAct molecular interaction database (IntAct)^36^, the Molecular INTeraction database (MINT)^37^, and functional protein association networks (STRING)^38^. The nodes in the network model were arranged into the above pathways based on the information in the KEGG pathway database.

### Statistical analysis

All values are presented as the mean ± S.E.M. Statistical analyses were determined using one-way analysis of variance (ANOVA) followed by Tukey’s multiple comparison tests. The value of statistical significance was set at *P *<* 0.05, **P *<* 0.01, or ***P *<* 0.001.

## Results

### Combination of four TFs is critical to establish iMSCs

To induce the direct conversion of fibroblasts to myogenic stem cells, we selected ten TFs, MyoD, Lin28, Pax3, Eya1, Bmi1, Esrrb, Lbx1, Ezh2, Dppa2 and Six1, that are known to be important for myogenic development and stem cell function and tested the function of the TFs in the direct reprograming of myogenic stem cells from MEFs. Among them, we found the combination of four master TFs (that will be noted 4F), Six1, Eya1, Esrrb and Pax3, reprogramed MEFs into myogenic stem cells.

**Fig. 1:**
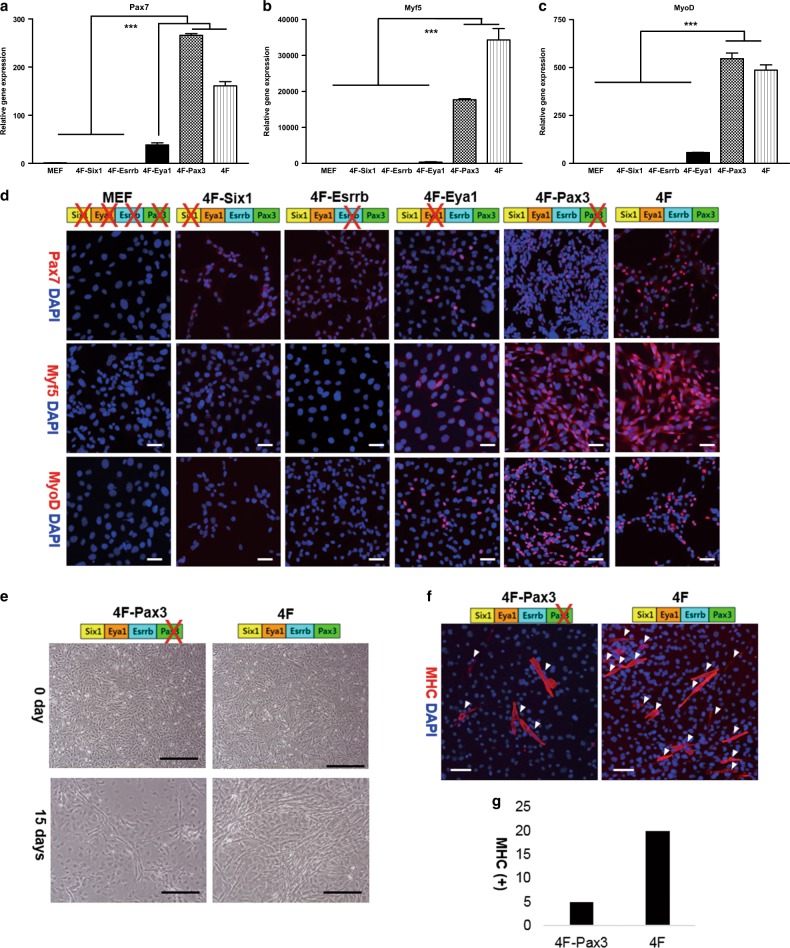
Comparison of expression levels of myogenic factors in N-1 study. **a**–**c** Real-time RT-PCR of Pax7, Myf5, and MyoD. **d** IF against Pax7, Myf5, and MyoD in N-1 study. Scale bar = 40 µm. **e** Phase contrast images of 4F-Pax3 and 4F before and after incubation in differentiation media for 15 days. Upper scale bar = 250 µm. Lower scale bar = 500 µm. **f** IF against MHC in 4F-Pax3 and 4F after incubation in differentiation media for 15 days. Scale bar = 40 µm. White arrow heads indicate MHC-positive muscle fibers. Each myogenic marker is represented in red. Nuclei are shown in blue stained by DAPI. **g** The number of MHC-positive cells in 4F-Pax3 and 4F after incubation in differentiation media for 15 days. One-way ANOVA was used for statistical analysis. Data are shown as the mean ± S.E.M. (***P *<* 0.001)

To identify key TFs that reprogram MEFs into myogenic stem cells among the four TFs, we transduced MEFs with only three of the four TFs: 4F-Six1 represented cells transduced with four transcriptional factors minus Six1 (Eya1, Esrrb, Pax3), 4F-Esrrb represented cells transduced with four transcriptional factors minus Esrrb (Six1, Eya1, Pax3), 4F-Eya1 represented cells transduced with four transcriptional factors minus Eya1 (Six1, Esrrb, Pax3) and 4F-Pax3 represented cells transduced with four transcriptional factors minus Pax3 (Six1, Eya1, Esrrb). We evaluated the expression level of myogenic factors of the transduced cells by real-time PCR and IF. In real-time PCR analysis, 4F-Six1 and 4F-Esrrb showed extremely low expression of Pax7, Myf5 and MyoD, which were similar to those of MEFs grown in myogenic growth media. 4F-Eya1 also expressed low levels of myogenic genes. However, Pax7, Myf5 and MyoD were highly expressed in both 4F-Pax3 and 4F. Additionally, 4F-Pax3 showed higher expression than 4F, except for Myf5 (Fig. 1a–c).

These real-time RT-PCR results were coincided with those of IF. According to the IF of Pax7 and MyoD, no or only a few Pax7- and MyoD-positive cells were observed in 4F-Six1 (Pax7 0%, Myf5 0%, MyoD 0%), 4F-Esrrb (Pax7 0%, Myf5 0%, MyoD 0%), and 4F-Eya1 (Pax7 8%, Myf5 10%, MyoD 16%). However, both 4F-Pax3 and 4F had a lot of positive cells for Pax7, Myf5, and MyoD. However, both 4F-Pax3 and 4F showed large numbers of cells positive for Pax7, Myf5, and MyoD. 4F-Pax3 showed 42% of Pax7-positive cells and 57% of MyoD-positive cells. 4F showed 31% of Pax7 positivity and 35% MyoD positivity. 4F-Pax3 showed higher expression of Pax7 and MyoD compared to 4F. However, the expression of Myf5 was higher in 4F (62%) than in 4F-Pax3 (38%) (Fig. 1d).

Interestingly, 4F-Pax3 showed minimal differentiation into muscle fibers when incubated in myogenic differentiation media (DM) for 15 days (Fig. 1e), although early myogenic differentiation markers (Pax7, Myf5, and MyoD) were highly expressed in 4F-Pax3 (Fig. 1a–d). Most 4F-Pax3 cells died in 1 week during incubation in DM, while the viability of 4F was high under the same myogenic DM condition (Fig. 1e). Only a few surviving 4F-Pax3 cells elongated and formed myotubes in myogenic DM. We plate the same number of cells on 2 well chamber slides and wait until the confluency reached 90%. When the cell confluency reached 90%, we incubate each cells in myogenic differentiation media. Thus, the initial cell number of 4F and 4F-Pax3 was same. However almost 60% of the initial cells were disappeared during the medium exchange.

The 4F cells elongated and fused together to form myotubes in myogenic DM (Fig. 1e). After incubation in myogenic DM, 4F showed numerous MHC-positive cells, while 4F-Pax3 showed a few MHC-positive cells (Fig. 1f). Without Pax3, the transduced cells could not differentiate into muscle fibers, although Pax7, Myf5, and MyoD expressions were high, suggesting that the four factors are necessary for giving myogenic differentiation capacity.

### Establishment of iMSCs with myogenic potentials

**Fig. 2:**
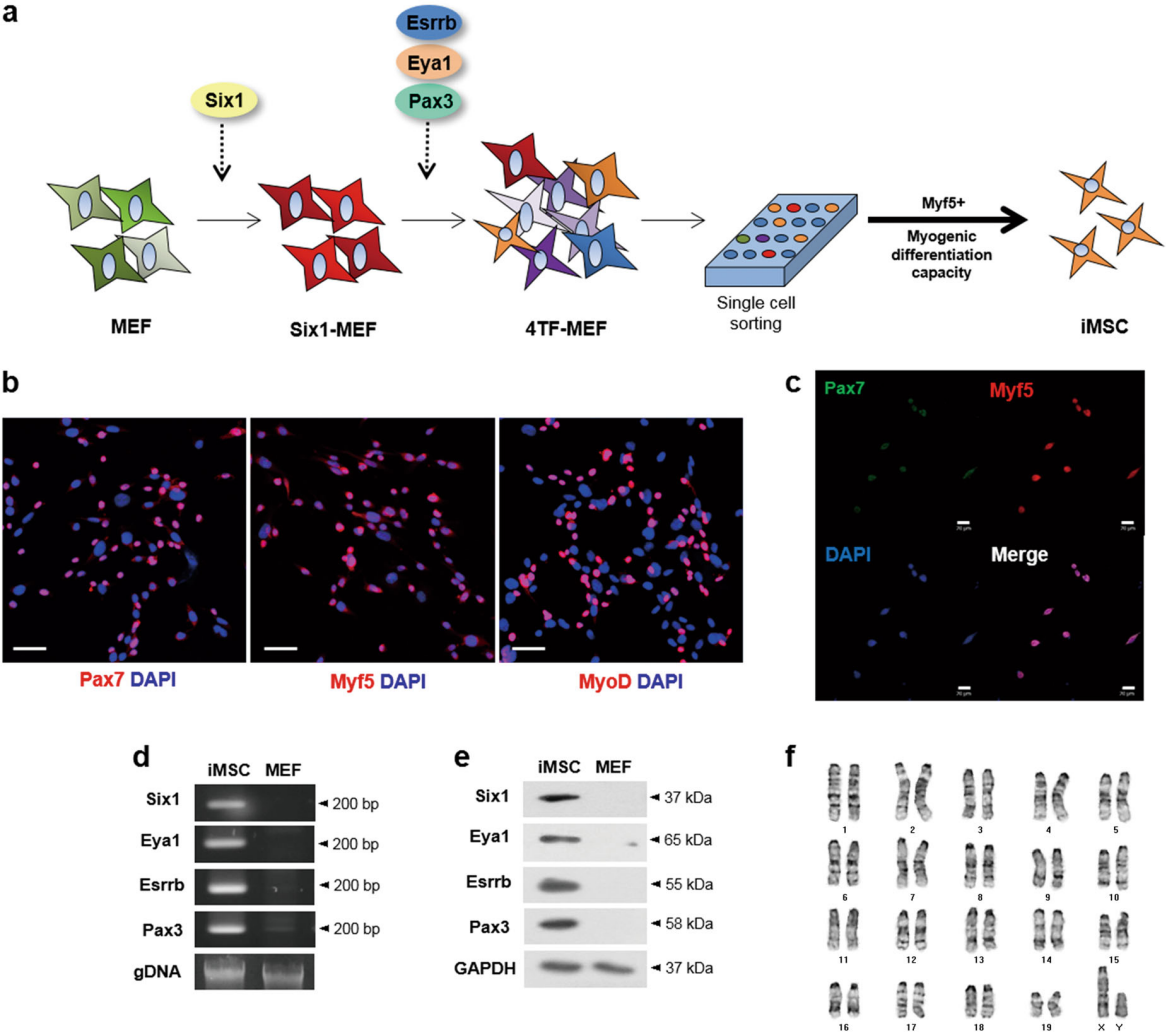
Establishment of iMSCs. **a** Scheme of establishment of iMSCs from mouse embryonic fibroblasts. MEF mouse embryonic fibroblast, Six1-MEF Six1 transduced MEFs, 4TF-MEF 4TFs (Six1, Eya1, Esrrb, and Pax3) transduced MEFs; iMSC induced myogenic stem cells. **b** IF of Pax7, Myf5, and MyoD. Each myogenic marker is represented in red. Nuclei are shown in blue stained by DAPI. Scale bar = 40 µm. **c** Co-staining of Pax7 and Myf5. Pax7 is represented in green and Myf5 is represented in red. Scale bar = 20 µm. **d** Genome integration test. All the size of the bands is 200 bp. The gel images were cropped and aligned. **e** Immunoblotting of transduced factors—Six1 (37 kDa), Eya1 (65 kDa), Esrrb (55 kDa), Pax3 (58 kDa), and GAPDH (37 kDa). The blot images were cropped and aligned. All the four factors were expressed in protein level. **f** Karyotype analysis of iMSCs. The iMSCs showed a normal karyotype (38, XY)

After transduction of the four TFs, we isolated single-cell colonies, since it is hard to distinguish myogenic stem cells from MEFs by morphology. Among the 99 single-cell lines, we sorted out 22 single-cell lines showing high expression of both Pax7 and Myf5. To clarify the myogenic differentiation capacity in vitro, we incubated each single-cell line in myogenic differentiation media. The nine single-cell lines among the 22 of Myf5 + lines represented high myogenic differentiation capacity showing multinucleated muscle fibers. We refer the cells as induced myogenic stem cells (iMSCs). The established iMSCs were positive for Pax7 (55.83%), Myf5 (44.53%), and MyoD (56.28%) in IF (Fig. 2b). And all the Pax7-positive cells were positive for Myf5 (Fig. 2c). The four TFs were integrated stably into the genome of iMSCs and expressed at the protein level (Fig. 2d, e). The karyotype of iMSCs was normal (Fig. 2f).

### iMSCs are more proliferative than MDSCs and MEFs

**Fig. 3:**
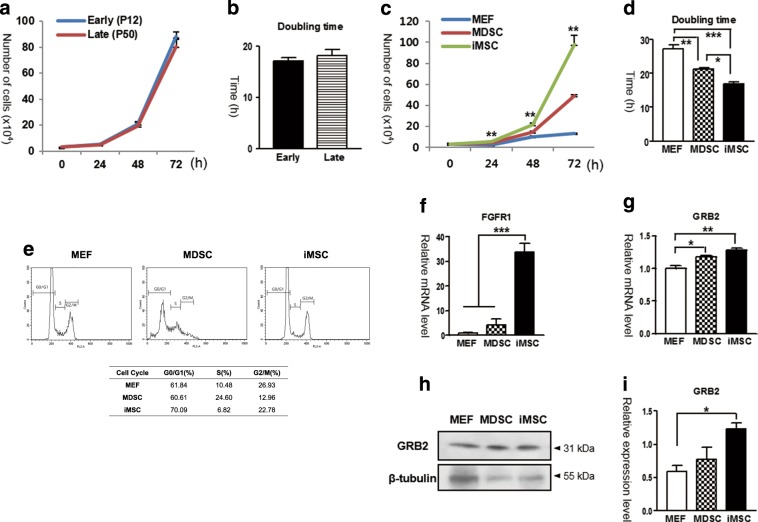
Proliferation capacity of iMSCs. **a** Cell growth curve of early and late passages of iMSCs. Early passage = P12, Late passage = P50. **b** Doubling time of early and late passages of iMSCs. Early passage = P12, Late passage = P50. **c** Cell growth curve of MEFs, MDSCs, and iMSCs. **d** Doubling time of MEFs, MDSCs, and iMSCs. **e** Cell cycle analysis using FACS. **f**, **g** Gene expression levels of FGFR1 and GRB2 measured by real-time RT-PCR. **h**, **i** Immunoblotting of GRB2 (31 kDa). Normalization was performed with ß-tubulin (55 kDa). The blot images were cropped and aligned. Image J was used for quantification of immunoblotting bands. One-way ANOVA was used for statistical analysis followed by Tukey’s multiple comparison test (***P *<* 0.001, **P < 0.01). Data are shown as the mean ± S.E.M. P passage

Prolonged proliferation of stem cells in vitro is a key feature for the therapeutic purpose of stem cells. We, therefore, measured the proliferation capacity of iMSCs. When we compared the cell growth curve of iMSC at early (P12) and late (P50) passages, iMSCs at each two passages showed no difference in their proliferation capacity (Fig. 3a). The doubling time also had no significant difference between the two passages (*p* = 0.322), suggesting that the iMSCs are stably expandable without losing their proliferative capacity though the passage number is increased (Fig. 3b).

When we compare the proliferation capacity of iMSCs to that of MDSCs and MEFs, the iMSCs showed higher proliferation capacity than the others. To measure the proliferation capacity, we seeded 3 × 10^4^ of MEFs, MDSCs, and iMSCs in each well and counted the number of cells every 24 h. After 72 h of incubation, the number of iMSCs was 98.25 × 10^4^ ± 8.66, while the numbers of MEFs and MDSCs were 12.92 × 10^4^ ± 5.83 and 49.17 × 10^4^ ± 2.79. Thus, these data indicate that iMSCs have higher proliferation capacity than MDSCs and MEFs (Fig. 3c). Additionally, the doubling time of iMSCs (16.76 h) was shorter than MEFs (27.08 h) and MDSCs (21.16 h) (Fig. 3d).

When we evaluated the cell cycle of iMSCs, 70.09% of iMSCs were at G0/G1 phase, which was higher than the proportions of MDSCs (60.61%) and MEFs (61.84%) at G0/G1 phase, whereas the proportions of iMSCs at the S phase and G2/M phase were 6.82% and 22.78%, respectively, which were relatively low (Fig. 3e).

To investigate a high proliferation capacity of iMSCs, we evaluated the expression of fibroblast growth factor receptor1 (FGFR1) and growth factor receptor bound protein 2 (GRB2) in iMSCs. According to real-time RT-PCR, iMSCs showed significantly higher expression of FGFR1 than MEFs and MDSCs (***P *<* 0.001) (Fig. 3f). Also, iMSCs showed higher mRNA and protein levels of GRB2 than MEFs and MDSCs (Fig. 3g–i).

### More myogenic iMSCs can be further enriched by CD106-negative and α7-integrin-positive fraction

**Fig. 4:**
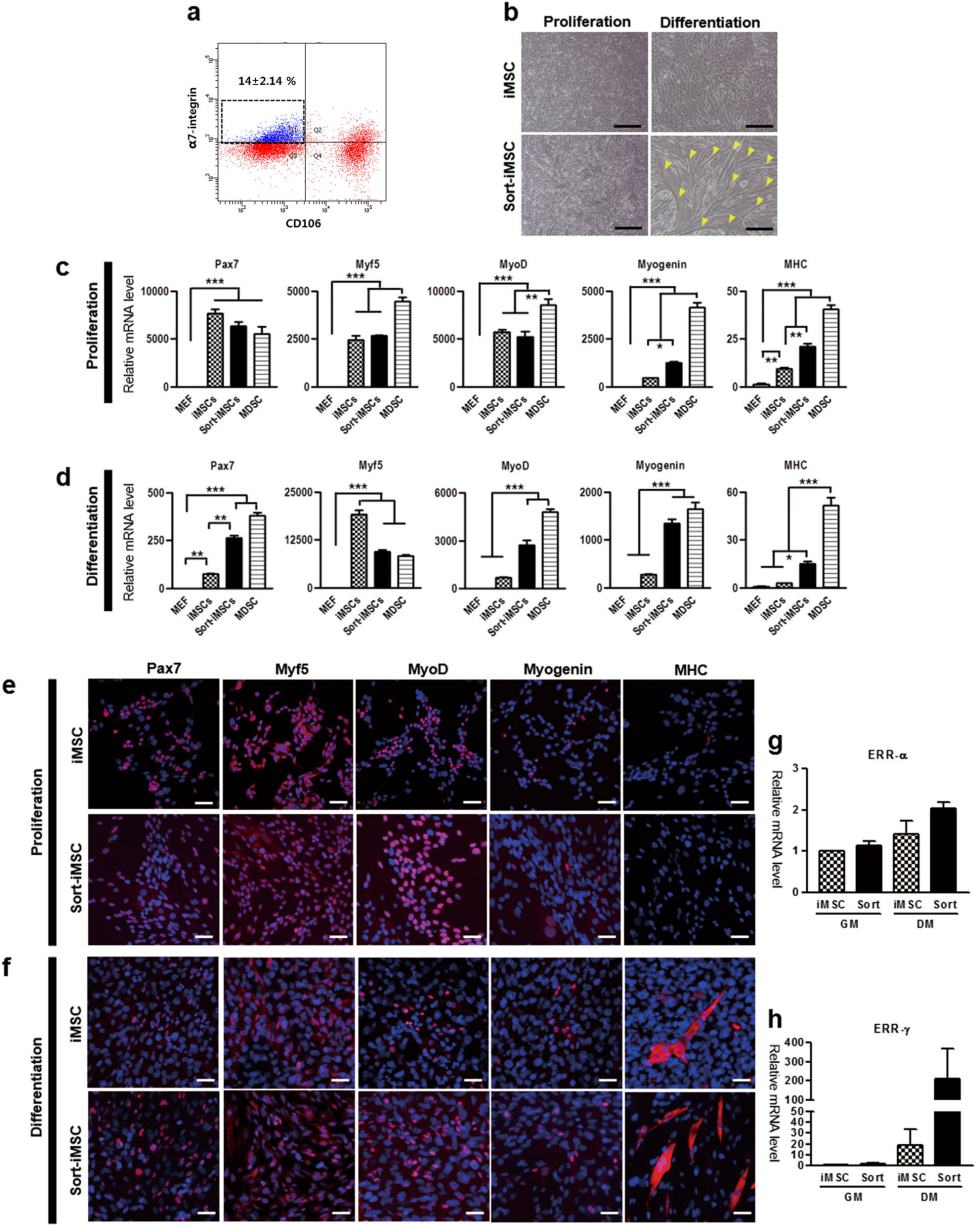
Comparison of iMSCs and sort-iMSCs in both GM and DM. **a** FACS sorting of iMSCs using CD106(–) and α7-integrin(+). **b** Phase contrast images of iMSCs and sort-iMSCs. iMSCs and sort-iMSCs were maintained in proliferation or myogenic differentiation media. Yellow arrows indicate multinucleated fibers, indicating late-stage muscle differentiation in vitro. Scale bar = 500 µm. **c** Gene expressions of MEF, MDSCs, iMSCs, and sort-iMSCs under GM. **d** Gene expressions of MEF, MDSC, iMSCs, and sort-iMSCs under DM. **e** IF for Pax7, Myf5, MyoD, myogenin, and MHC of iMSCs and sort-iMSCs in GM. **f** IF for Pax7, Myf5, MyoD, myogenin, and MHC of iMSCs and sort-iMSCs in DM. Each myogenic marker is represented in red. Nuclei are shown in blue stained by DAPI. Scale bar = 40 µm. **g**, **h** Expression levels of ERR-α and ERR-γ of iMSCs and sort-iMSCs in GM and DM measured by real-time RT-PCR. Data are shown as the mean ± S.E.M (***P *<* 0.001, **P < 0.01, *P < 0.05). GM growth media, DM differentiation media

To enhance myogenic differentiation capacity, we sorted the iMSCs with the cell surface markers, CD106 and α7-integrin, based on the FACS analysis results (Fig. S1). A subset of iMSCs was found to be CD106 ( - ) and α7-integrin ( + ) (14 ± 2.14 %) (Fig. 4a). We referred to these as sort-iMSCs. When we maintained the iMSCs and sort-iMSCs in growth media, the sort-iMSCs were more refractive and smaller than iMSCs. In 1 day after incubation of sort-iMSCs in myogenic DM, the cells began to elongate and fuse together, causing the nucleus to come into line. In 3 days after incubation in myogenic DM, sort-iMSCs formed multinucleated branch-shaped myotubes. The iMSCs also showed such differentiation in myogenic DM, but only a limited differentiation to few multinucleated myotubes, which are a late myogenic differentiation marker (Fig. 4b).

To clarify the myogenic differentiation capacity of iMSCs and sort-iMSCs, we compared the expression of Pax7, Myf5, MyoD, myogenin, and MHC under both proliferation and differentiation conditions. Both iMSCs and sort-iMSCs showed high expression levels of Pax7 comparable to MDSCs in proliferation media. Both iMSCs and sort-iMSCs showed higher expression of Myf5, MyoD, myogenin, and MHC in proliferation media than MEFs, but lower expression than MDSCs (Fig. 4c–e). On the other hand, sort-iMSCs showed higher expression of myogenin and MHC than iMSCs, although iMSCs and sort-iMSCs showed similar expression of Pax7, Myf5, and MyoD (Fig. 4c). We maintained the cells in myogenic DM for 3 days. In 3 days after incubation in DM, sort-iMSCs showed higher expression of all the myogenic regulatory genes except Myf5 compared to iMSCs. Additionally, sort-iMSCs showed comparable expression of Myf5 and myogenin to MDSCs in DM (Fig. 4d–f). Additionally, sort-iMSCs showed comparable expression of Myf5 and myogenin to MDSCs in DM (Fig. 4d–f). Taken together, iMSCs showed upregulation of early myogenic regulatory factors including Pax7, Myf5, and MyoD in proliferation media. In myogenic differentiation media, sort-iMSCs showed upregulation of late myogenic regulatory factors including myogenin and MHC. Thus, sort-iMSCs exhibited higher myogenic differentiation capacity than iMSCs. To clarify the reason for higher myogenic differentiation capacity of sort-iMSCs, we checked the expression of ERR-α and ERR-γ, known as regulators of myogenic differentiation through regulation of mitochondrial biogenesis in skeletal muscle^39^. ERR-γ and ERR-α in sort-iMSCs were upregulated in DM condition, compared to iMSCs (Fig. 4g, h). The increased expression of ERR-γ and ERR-α in sort-iMSCs indicates that sort-iMSCs are more potent in oxidation, leading to upregulation of myogenic regulatory factors, including myogenin, and myogenic differentiation.

### Sort-iMSCs restore more dystrophin than MDSCs and MEF in mdx mice

**Fig. 5:**
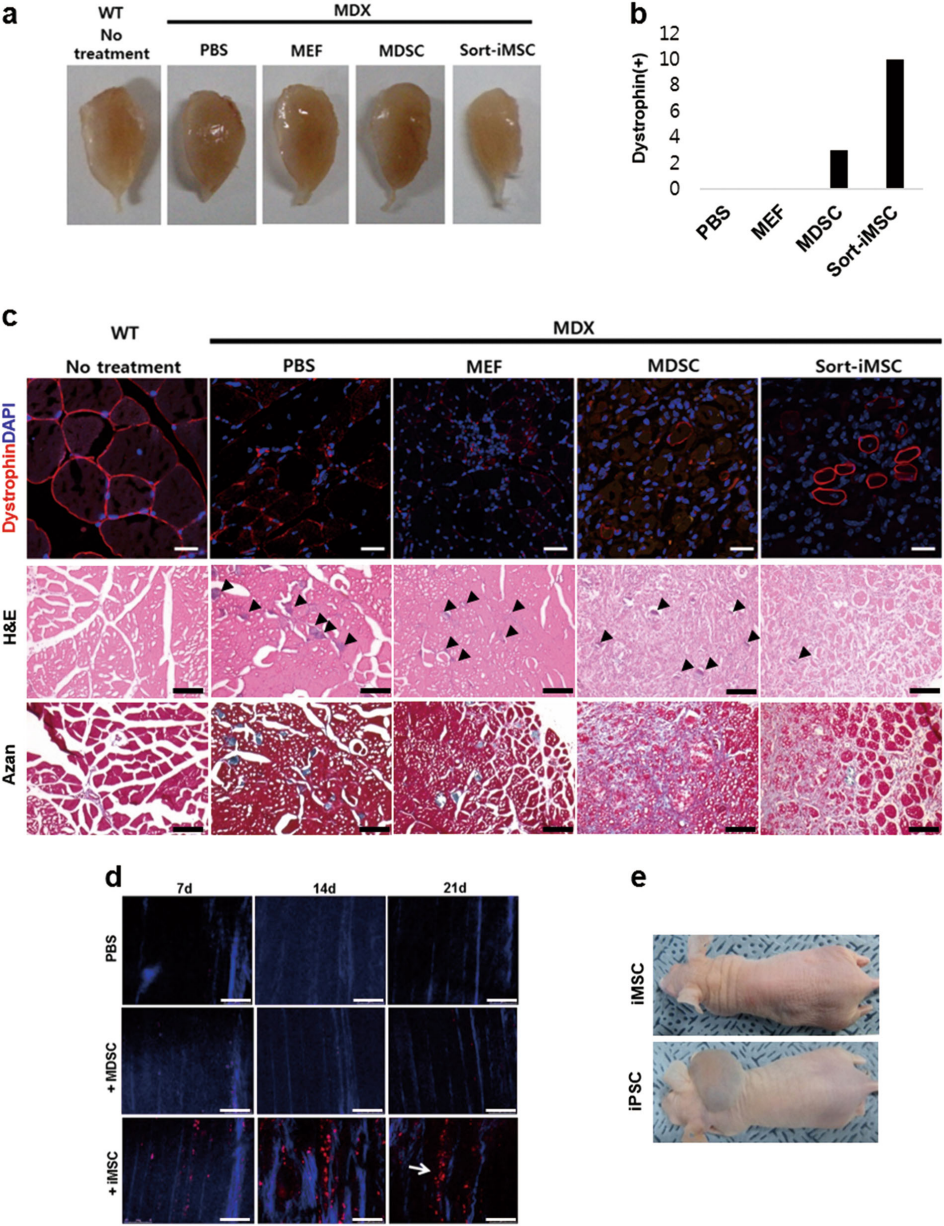
Myogenic differentiation and proliferation capacity in mdx mice. **a** In vivo differentiation capacity of sort-iMSCs. Gross observation of cell-transplanted T.A muscle of mdx mice. **b** The number of dystrophin-positive cells in IF staining against dystrophin. **c** IF staining against dystrophin (upper panel). Representative images of transverse section of T.A muscle. Dystrophins are represented in red. Nuclei are shown in blue stained by DAPI. Scale bar = 40 µm. Hematoxyline and eosin staining (middle panel). Arrow heads indicate calcification area. Scale bar = 100 µm. Azan staining (lower panel). Scale bar = 100 µm. **d** In vivo proliferation capacity of iMSCs. Sequential images of TA muscle repair transplanted with 1 × 10^5^ iMSCs or MDSCs at 7, 14, and 21 days after transplantation. Collagen fibrils of the muscle fiber are shown in blue. The transplanted cells are represented in red. Arrow indicates regenerating fiber cells. Scale bar = 75 µm. **e** Tumor formation test of iMSCs in nude mice. WT wild type, iPSC induced pluripotent stem cell

So far, we checked the characteristics of iMSCs and sort-iMSCs in vitro. We evaluated myogenic differentiation capacity of sort-iMSCs in vivo, since sort-iMSCs showed more potent myogenic differentiation capacity than iMSCs in vitro. To evaluate myogenic differentiation of sort-iMSCs in vivo, we injected the cells into mdx mice that do not express dystrophin. In 4 weeks after cell injection, the sort-iMSCs injected muscles were regenerated and appeared normal in gross findings without any neovascularization and granulation tissue (Fig. 5a). The sort-iMSCs transplanted-TA muscle contained dystrophin-positive muscle fibers, whereas either MEFs or MDSCs transplanted-TA muscle showed few or only a few dystrophin-positive fibers. These data indicate that the injected sort-iMSCs were engrafted successfully, survived in the injured muscle, and differentiated into muscle fibers. We confirmed that the myogenic differentiation capacity of sort-iMSCs was higher than MEFs and MDSCs in vivo (Fig. 5b, c). In H&E staining, we detected calcification area (arrow heads in Fig. 5c) in all the notexin injected-mdx mice except sort-iMSCs group. Reduced calcification area in sort-iMSCs group represents that more recovery of damaged muscle cells is achieved in sort-iMSCs group than other group (Fig. 5c). In Azan staining, unlike deep-blue stained MDSC injected group, the sort-iMSCs injected group showed weak stainability representing less collagen fibers produced in sort-iMSCs injected group. None of the injected mice showed apparent immune reactions and infiltration of inflammatory cells (Fig. 5c).

### iMSCs are more proliferative than MDSCs in mdx mice

To evaluate the early engraftment and proliferation of iMSCs in vivo, we transplanted Tomato + iMSCs (5 × 10^4^ cells/mouse) or iRFP + MDSCs (5 × 10^4^ cells/mouse as a control) into injured muscles and track them in vivo real-time by using an *intravital* imaging system sequencially. At 7 days after transplantation, the Tomato + iMSCs engrafted successfully and aligned with exiting fibers were observed. Sequential *intravital* imaging at 14 and 21 days after transplantation revealed that the number of transplanted tomato + iMSCs was increased and they incorporated into regenerating fibers at 21 days, indicating iMSCs are transplantable and retain their myogenic ability even after transplantation in vivo in injured muscle. The engraftment efficiency and in vivo proliferation capacity were much higher in iMSCs compared to in MDSCs (Fig. 5d). Because of the high proliferation capacity of iMSCs in vivo, we evaluated whether the iMSCs have tumorigenicity. No tumor mass was observed in iMSCs injected mice, whereas iPSC-injected nude mice showed teratoma in 3 weeks (Fig. 5e). We confirmed that the iMSCs did not induce tumor formation when injected in vivo.

### Genome-wide mRNA expression analysis of iMSCs and sort-iMSCs

**Fig. 6:**
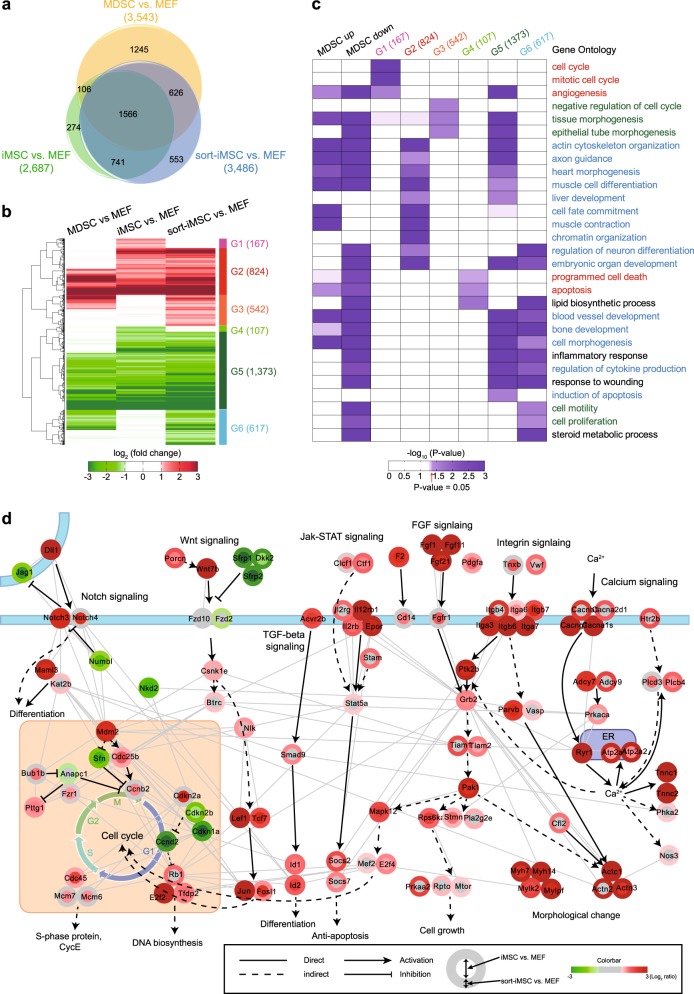
Comparison of genome-wide mRNA expression of MDSCs, MEFs, iMSCs, and sort-iMSCs. Differentially expressed genes in iMSCs and sort-iMSCs and their associated cellular processes and signaling pathways. **a** Relationship between the DEGs from the three comparisons, MDSCs vs. MEFs (3543 DEGs), iMSCs vs. MEFs (2687 DEGs), and sort-iMSCs vs. MEF (3486 DEGs). **b** Differential expression patterns of the DEGs in the six groups (G1-6). Numbers in parenthesis denote the sizes of the DEGs in the corresponding groups. Colors in the heat map represent up- (red) and downregulation (green) of the genes (rows in the heat map). Color bar, gradient of log_2_-fold-changes of DEGs in the three comparisons. The dendrogram was generated by performing a hierarchical clustering of the log_2_-fold-changes of DEGs in the three comparisons (Euclidean distance as a dissimilarity measure and complete linkage). **c** GOBPs represented by the DEGs in G1-6, as well as upregulated (MDSCs up) and downregulated (MDSCs down) genes in MDSCs, compared to MEFs. Color gradient represents the significance, -log_10_(*P*-value), of the corresponding GOBPs being enriched by the DEGs in G1-6, MDSC up and MDSC down, where *P*-value is the enrichment *P*-value computed by DAVID. Font colors of GOBP labels represent groups of GOBPs: red, cell cycle-related processes represented by DEGs in iMSCs, but not by DEGs in sort-iMSCs; green, differentiation-related processes represented by DEGs in sort-iMSCs, but not by DEGs in iMSCs; and blue, cell cycle and differentiation-related processes represented by DEGs in both iMSCs and sort-iMSCs. **d** Signaling network model associated with proliferation and differentiation of iMSCs and sort-iMSC cells. Node and node border colors represent log_2_-fold changes of their corresponding genes in iMSCs vs. MEFs and sort-iMSCs vs. MEFs, respectivly. Color gradient represents log_2_-fold-changes of DEGs in the comparisons indicated in the legend (box at bottom right). The edges represent direct activation (arrows), repression (inhibition symbols), indirect activation through intermediate molecules not shown (dotted line) and PPIs (gray solid line), respectively. The types of interactions were obtained from the KEGG pathway database. Plasma membrane is denoted as the thick blue lines

To understand the molecular nature underlying differentiation and proliferation capacity of iMSCs and sort-iMSCs, we performed genome-wide gene expression profiling of MDSCs, MEFs, iMSCs, and sort-iMSCs using the Agilent SurePrint G3 mouse GE 8 × 60 K microarray. Using the gene expression profiles, we identified a total of 5,111 differentially expressed genes (DEGs) from the following three comparisons: (1) MDSC vs. MEF (3,543 DEGs with 1560 upregulated and 1983 downregulated), (2) iMSCs vs. MEF (2,687 DEGs with 1,064 upregulated and 1,623 downregulated), and (3) sort-iMSCs vs. MEF (3,486 DEGs with 1,382 upregulated and 2,104 downregulated) (Fig. 6a and Supplementary Table S1). To systematically explore these DEGs, we categorized them into 23 clusters (C1-23; Supplementary Table S2) based on their up- and downregulation in the tree comparisons. Of them, we focused on 10 clusters (C1 and C4-12) showing differential expression in the two comparisons of iMSCs vs. MEFs and sort-iMSCs vs. MEFs. These 10 clusters were further grouped into six groups (G1-6) based on their up- and downregulation patterns in the two comparisons (Fig. 6b and Supplementary Table S1). G1 and G5 comprised clusters up- and downregulated in iMSCs, respectively, but not in sort-iMSCs, compared to MEFs, whereas G3 and G6 comprised clusters up- and downregulated in sort-iMSCs, respectively, but not in iMSCs. G2 and G5 comprised clusters up- and downregulated commonly in both iMSCs and sort-iMSCs, respectively.

To understand the cellular processes associated with G1-6, we performed the enrichment analysis of GOBPs for the genes in G1-6 using DAVID software (Fig. 6c and Supplementary Tabls S3). G1 upregulated in iMSCs, compared to MEFs, significantly (*P* *<* 0.05) represented cell cycle-related processes (cell cycle and mitotic cell cycle), wherease G4 downregulated in iMSCs represented apoptosis-related processes (programmed cell death and apoptosis). Interestingly, of these processes, only the apoptosis-related processes were represented by the downregulated genes in MDSCs, compared to MEFs, but the cell cycle-related were represented by neiether the up- nor downregulated genes in MDSCs. Collectively, these data suggest that iMSCs show higher proliferation capacity than MEFs and even than MDSCs, consistent to our findings in Fig. 3c, d. Moreover, G3 and G5 up- and downregulated in sort-iMSCs, respectively, compared to MEFs, represented differentiation-related processes (tissue morphogenesis, epithelial tube morphogenesis, blood vessel development, bone development, and cell morphogenesis). All of these processes were also represented by the DEGs in MDSCs, compared to MEFs. These data suggest that sort-iMSCs show differentiation capacity compatible to MDSCs, which is consistent with our findings shown in Fig. 4d. Moreover, two differentiation-related processes (tissue and epithelial tube morphogenesis) were represented by G3, but by neither G1 nor G2, consistent to enhanced differentiation capacity of sort-iMSCs compared to iMSCs, consistent to our finding in vitro shown in Fig. 4.

### Network model describing signaling pathways associated with enhanced proliferation and differentiation capacity of iMSCs and sort-iMSCs

To examine the intracellular signaling pathways associated with proliferation and differentiation capacity of iMSCs and sort-iMSCs, we first selected the DEGs involved in the aforementioned cell cycle- and differentiation-related cellular processes represented by G1-G6 and then identified KEGG signaling pathways represented by these DEGs. Next, we reconstructed a network model describing the interactions among the DEGs involved in these signaling pathways (Fig. 6d). First, Notch and TGF-β signaling pathways were activated in iMSCs and sort-iMSCs, as indicated by upregulation of Notch3/4, Dll1, and Maml3 (Notch signaling) and Acvr2b, Smad9, and Id1/2 (TGF-β signaling) in iMSCs or sort-iMSCs, compared to MEFs. Second, Wnt and Jak-stat signaling pathways were activated in iMSCs and sort-iMSCs as indicated by upregulation of Wnt7b, Porcn, CsnK1e, Btrc, Lef1, and Tcf7 (Wnt signaling) and Il2rg/b, Il12rb1, Stam, Stat5a, and Socs2/7 (Jak-stat signaling) in iMSCs or sort-iMSCs, compared to MEFs. These pathways are known to be involved in cell proliferation and/or differentiation, suggesting their contirubtion to high proliferation capacity of iMSCs (Fig. 3c) and high differentiation capacity of sort-iMSCs (Fig. 5b). Third, FGF and integrin signaling pathways were activated in iMSCs and sort-iMSCs as indicated by upregulation of Fgf1/11/21 and Fgfr1 (Fgf signaling) and Itga3/6/7 and Itgb4/6/7 (Integrin signaling), as well as upregulation of downstream signaling molecules in the actin reorganization pathway (Ptk2b, Grb2, Tiam1/2, Pak1, Parvb, and Vasp) in iMSCs or sort-iMSCs, compared to MEFs. Finally, calcium signaling was activated in iMSCs and sort-iMSCs as indicated by upregulation of calcium transporters (Cacna1s/a2d1/b1/g1, Ryr1, and Atp2a1/2). The FGF, integrin, and calcium signaling pathways are known to activate myosins (Myh7/14 and Mylk2/pf), actins (Actc1/n2/n3) and troponins (Tnnc1/2), suggesting their contribution to morphological changes required for proliferation of iMSCs and differentiation of sort-iMSCs. Collectively, the network model suggests that these signaling pathways can play key roles in conferring higher proliferation and differentiation capacity to iMSCs and sort-iMSCs, respectively.

## Discussion

In this study, we established induced myogenic stem cells (iMSCs) by ectopic expression of Six1, Eya1, Esrrb, and Pax3. The iMSCs have myogenic differentiation capacity both in vitro and in vivo. Also, the iMSCs represented higher proliferative capacity even than MDSCs. The iMSCs indeed do not lose proliferative capacity until the passage was up to 50 (Fig. 3a). The ectopic expression of four TFs upregulates endogenous myogenic regulatory genes.

To clarify the role of the four TFs in the establishment of iMSCs, we transduced MEFs with three transcriptional factors omitting one of the four factors. Six1 and Esrrb play a pivotal role in converting MEFs to myogenic stem cells. Without six1 or esrrb, the increase of pax7, myf5, and myoD was minimal and establishment of iMSCs was hard to achieve (Fig. 1a–d). It is studied that Pax3 and Pax7 are key factors that confer early myogenic capacity leading to myotome during mouse muscle development^23^. However, overexpression of Pax3, without Six1 or Esrrb, is not sufficient to upregulate Pax7 and convert MEFs to myogenic lineage cells in 4F-Six1 and 4F-Esrrb.

The expression of myogenic factors was slightly increased in 4F-Eya1. Eya1 has dual role as a protein tyrosine phosphatase and transcriptional cofactor^40^. Eya1 acts as phosphatase and turns on Six1 from repression to activation^19,40^. Without Eya1, Six1 cannot function as much as interacting with Eya1. The interaction of Six1 and Eya1 directly activates Pax3 in limb muscle development, suggesting that the expression of Six1 and Eya1 increase the expression of Pax3^18,19^.

Interestingly, 4F-Pax3 cells represented higher expression in Pax7 than 4F cells, while similar expression was observed for MyoD and lower for Myf5 (Fig. 1a–d). The increase of Pax7 in 4F-Pax3 may be caused by the interaction of Six1 and Eya1. However, without Pax3, the cells could not differentiate into myotubes, even when myogenic expressions were increased (Fig. 1). Pax7, a paralogue of Pax3, can substitute for Pax3 because it shares most myogenesis functions with Pax3, including the regulation of MyoD^41^. Although the increased expression of Pax7 in 4F-Pax3 can replace ectopic Pax3 in terms of regulating myogenic factors including Myf5 and MyoD under the proliferation condition, it is not enough to trigger myogenic differentiation.

The increased expression of Pax7 may explain the insufficiency of muscle differentiation of 4F-Pax3 in the differentiation environment (Fig. 1e, f). For muscle differentiation, Pax7 expression must be downregulated^42^. Activated satellite cells undergo sequential processes of proliferation, downregulation of Pax7, and then differentiation into muscle fibers. Pax7 activates proliferation of satellite cells but delays the expression of myogenin leading to delays of differentiation^42^. Particularly, during Myf5 expression, which is the determinant of myogenesis, 4F cells shows higher expression level than 4F-Pax3, as Myf5 is the direct target of Pax3^24^. To differentiate into muscle fibers, the cells needs proper cell number and confluency to connect adjacent cells. The myogenic differentiation media contains minimum nutrients and the only the cells with myogenic differentiation potential can be survived in that such a harsh niche. The cells that do not survive in the myogenic differentiation medium, like 4F-Pax3, are cells that lack the ability to differentiate into muscle functionally.

Thus, all the four factors are necessary to induce myogenic stem cells, which have myogenic differentiation capacity in DM not only express myogenic factors. Without one of the four TFs, MEFs could not convert into myogenic stem cells which express myogenic regulatory factors and differentiate into myogenic fibers under differentiation condition. Various combinations of candidate TFs can be used to induce direct conversion into myogenic stem cells. Naoki et al.^14^ suggested that the combination of six transcription factors induce direct reprogramming of skeletal muscle progenitors from embryonic fibroblasts. However, the combination of TFs for direct conversion should be accurate because it is very delicate. Induction with or without one specific TF can trigger reprogramming of totally different type of cells. For example, without Pax3, in this study, the cells represented proliferative capacity with weak myogenic differentiation. Thus, accurate combination of TFs is important to establish myogenic stem cells with both proliferation and myogenic differentiation capacities.

The established iMSCs with the four factors represented high expression levels of early myogenic regulatory factors including Pax7, Myf5, and MyoD (Fig. 2b). Interestingly, iMSCs represent even 1.38 times higher Pax7 expression than MDSCs and 7,634 times higher than MEF (*P* < 0.001). Compared with the fact that <30% of MDSC is Pax7-positive, about 55.83% of iMSCs are positive for Pax7, which is quite high expression^43^. Pax7 promotes self-renewal of satellite cells and maintains the satellite cell pool^44^. It is revealed in Pax7 mutant mice that Pax7 have anti-apoptotic function^41^. Also, repressed expressions of late differentiation markers including myogenin and MHC enhanced the proliferation of iMSCs under proliferation condition. Thus, the increased expressions of early myogenic factors are related to the high proliferation capacity of iMSCs. Additionally, iMSCs are expandable up to passage 90 (data not shown). This is a major difference between iMSCs and MDSCs. The low expressions of myogenin and MHC maintain the elevated expression levels of Pax7 and Myf5 and proliferation capacity in GM condition.

In the FACS analysis, the iMSCs showed distinct expression patterns with MDSCs except for α7-integrin. The iMSCs were strongly positive for α7-integrin (97.23 ± 0.43 %) which is compatible to satellite cells. α7-integrin is one of the most well-known cell surface markers of satellite cells and myoblasts^45^. α7-integrin can be used to distinguish myoblasts from fibroblasts^46^. Te iMSCs were intermediately positive for CD34 (14.20 ± 2.92%) and Syndecan 4 (7.57 ± 3.56%) (Fig. S1). Satellite cells are positive for α7-integrin, CD34, CXC motif reception R-4 (CXCR) and CD106 and negative for CD45, CD31, CD11b, and Sca-1^47–49^. The iMSCs can be distinguished from satellite cells based on their reactivity against CXCR, CD34, CD106, and Sca-1. Although iMSCs showed myogenic differentiation capacity, the cells differed from satellite cells in terms of cell surface markers.

Interestingly, the FACS analysis of iMSCs revealed two distinct peaks for CD106, Sca-1, and CD73, indicating that the cells were composed of two populations (Fig. S1). All the three markers are mesenchymal stem cell markers^50,51^, suggesting that the cells contained both mesenchymal stem cell stage- and non-mesenchymal stem cell stage-populations. We revealed that sorting with α7-integrin and the absence of CD106 increased both the myogenic differentiation capacity and myogenic regulatory factor gene expressions (Fig. 4). It has been revealed that FACS sorting with specific cell surface markers enhances the myogenic differentiation capacity^52^.

We compared the myogenic capacity of iMSCs and sort-iMSCs under both proliferation and differentiation condition. The iMSCs exhibited a spindle-shaped cytoplasm and round nucleus, which were similar to mesenchymal stem cells in proliferation media. The sort-iMSCs showed robust myogenic differentiation capacity when incubated in myogenic differentiation media for 3 days (Fig. 4b). In the myogenic differentiation media, the sort-iMSCs represented higher expressions of myogenic regulatory factors than iMSCs, excepting for Myf5 (Fig. 4d). Particularly, myogenin expression in sort-iMSCs increased significantly, comparable to MDSCs. Downregulation of Myf5 in sort-iMSCs may lead to upregulation of late myogenic differentiation factors including MyoD, myogenin, and MHC in DM. Thus, the sort-iMSCs may be more reactive to the myogenic differentiation niche than iMSCs, considering that they share similar myogenic expression pattern in proliferation media (Pax7, Myf5, and MyoD in Fig. 4c), but completely different expression in differentiation media (Fig. 4d). It suggests that the sort-iMSCs have greater myogenic differentiation capacity than iMSCs. Thus, we obtain enough number of iMSCs in myogenic growth media and sort the cells using the cell surface markers when we apply in vivo test or check myogenic differentiation capacity.

The high myogenic differentiation ability of sort-iMSCs may be related to its increased expression of ERR-γ and ERR-α in DM (Fig. 4g, h). In the previous study, muscle specific ERR-γ –/– represented immature myotube formation^39^. It is revealed that increase of mitochondrial biogenesis is needed to induce myogenic differentiation^53^ and ERR-γ increased mitochondrial activity and oxidative capacity in skeletal muscle^54^. And ERR-α regulates myogenic differentiation cooperating with PGC-1α, which is one of master regulator of mitochondrial biogenesis in skeletal muscle^55–57^.

We evaluated the myogenic differentiation properties of sort-iMSCs in vivo. The sort-iMSCs differentiated into myotubes and expressed dystrophin when transplanted into mdx mice (Fig. 5a–c). It suggests that the sort-iMSCs can be successfully engrafted, survive, and differentiate into myotubes in vivo. Although satellite cells injected-mdx mice showed a few dystrophin-positive fibers, the number was much smaller than sort-iMSCs injected one. Sort-iMSCs injection restored more dystrophin (+) fibers than MEFs or MDSCs in mdx mice indicating that the sort-iMSCs are more potent than MEFs and even MDSCs when transplanted in vivo (Fig. 5c). In most studies, researchers destroy the endogenous satellite cells of the recipient mdx mice through the 18G irradiation, and then transplant satellite cells or myoblasts into the muscle of recipient^58,59^. This can avoid competition between endogenous satellite cells and exogenous muscle stem cells, and maximize the engraftment efficiency of transplanted cells. However, in the case of depleting satellite cells by irradiation, it cannot be said that the experiments in which the intramuscular environment is reproduced since all the endogenous satellite cells have been destroyed. Therefore, in this study, we evaluated the ability of sort-iMSCs to survive competition with existing endogenous satellite cells without irradiation. Thus, the positive control, MDSCs, represent lower levels of dystrophin than in previous studies which deplete the endogenous satellite cells in mdx^47,60,61^. Consider that we do not irradiate the mdx mice, sort-iMSCs can be survived in the environment which have been already occupied by endogenous satellite cells.

We established induced myogenic stem cells through ectopic expression of Six1, Eya1, Esrrb and Pax3. In contrast, Ito et al. established the induced skeletal muscle progenitor cells (iSkMs) through ectopic expression of a transcriptional factors including Pax3^14^. There is a clear difference between induced muscle progenitor cells and induced muscle stem cells. The cell fates of muscle stem cell are proliferation and/or muscle differentiation, whereas the cell fate of muscle progenitor is muscle differentiation. In this study, we confirmed robust proliferation or iMSCs different from previous iSkMs, and it was confirmed that the proliferative capacity was maintained even after the passage, and that it was a muscle stem cell in which differentiated into muscle cells.

The proliferative capacity of iMSCs is superior to MDSCs. The proliferation capacity of MDSCs is low and the proliferation rate is getting slower as the passage number is increased, since they are primary cells. In the proliferation assay, iMSCs represent higher proliferation capacity than MDSCs both in vitro and in vivo (Figs. 3 and 5c). To reveal the involved mechanisms, we checked FGF signaling which is well-known for its function in cell proliferation^62^. Interestingly, the expression of FGFR1 was much higher than in both MEFs and MDSCs (***P < 0.001) (Fig. 3f). In myoblast, proliferation is decreased by the repression of FGFR1 through KLF10^63^. FGFR1 promotes the proliferation of stem cells through cyclin-dependent kinase inhibitors^64,65^. It is consistent to our cell cycle data that iMSCs showed high percentage of G0/G1 (Fig. 3e). In this study, the expression of GRB2 was increased in iMSCs (Fig. 3g–i). Further studies are needed to reveal the relationship between proliferation, GRB2, and FGF signaling.

Because of the use of monocystronic vectors, ectopic expression of many genes can create various cell populations. To exclude this possibility, single-cell sorting was performed in this experiment. Each cell line was derived from each cell. Although the iMSC is originated from a single-cell, the iMSC has a heterogeneous characteristic as it continues to pass, suggesting that the iMSC undergoes asymmetric division like satellite cell. This was confirmed by cell morphology and FACS analysis (Fig. S1).

In this study, we established stably expandable induced myogenic stem cells with four defined factors. Especially, the iMSCs have much higher proliferation capacity than MDSCs. The established cells have both myogenic differentiation capacity and robust proliferation properties. The iMSCs can differentiate and form myotubes in vivo without tumor formation. The stably expandable iMSCs established in this study provide new source for drug screening and muscle regenerative therapy.

## Electronic supplementary material

Supplementary material

Supplementary Figure S1

Supplementary Figure S2

Supplementary Figure S3

Supplementary Table S1

Supplementary Table S2

Supplementary Table S3
