# Supplementary material for: Establishment of stably expandable induced myogenic stem cells by four transcription factors
Source: Cell Death Dis. 2018 Oct 25;9(11):1092. doi: 10.1038/s41419-018-1114-8 (PMC6202407; doi:10.1038/s41419-018-1114-8)
Supplement: Supplementary file 1 — Supplementary material [file 41419_2018_1114_MOESM1_ESM.doc]

**Establishment of stably expandable induced myogenic stem cells by four transcription factors**

**Running head :** Stably expandable induced myogenic stem cells

Eun-Joo Lee^1,*^, Minhyung Kim^2,*^, Yong Deuk Kim^1,3^, Myung-Jin Chung^1,3^, Ahmed Elfadl^1,3^, HM Arif Ulah^1,3^, Hamilton Wang^4,5^, Dongsu Park^4,5,6^, Sunray Lee^7^, Hyun-Sook Park^7^, Tae-Hwan Kim^1^, Daehee Hwang^2,8,9^, and Kyu-Shik Jeong^1,3,#^

**Affiliation** : ^1^Department of Veterinary Pathology, College of Veterinary Medicine, Kyungpook National University, Daegu 41566, Republic of Korea, ^2^School of Interdisciplinary Bioscience and Bioengineering, Pohang University of Science and Technology, Pohang 37673, Republic of Korea, ^3^Stem Cell Therapeutic Research Institute, Kyungpook National University, Daegu 41566, Republic of Korea, ^4^Department of Molecular Human Genetics, Baylor College of Medicine, One Baylor Plaza, Houston, TX, 77030, USA, ^5^Center for Skeletal Biology, Baylor College of Medicine, One Baylor Plaza, Houston, TX, 77030, USA. ^6^Department of Pathology and Immunology, Baylor College of Medicine, One Baylor Plaza, Houston, TX, 77030, USA, ^7^Cell Engineering for Origin Research Center 45-13, Ujeongguk-ro, Jongno-gu, Seoul 03150, Republic of Korea, ^8^Center for Plant Aging Research, Institute for Basic Science, Daegu Gyeongbuk Institute of Science and Technology (DGIST), Daegu 42988, Republic of Korea, ^9^Department of New Biology, DGIST, Daegu 42988, Republic of Korea

*These authors contributed equally to this work.

**#Corresponding author**:

Kyu-Shik Jeong, D.V.M., Ph.D

E-mail: [jeongks@knu.ac.kr](mailto:jeongks@knu.ac.kr)

Telephone: 82-53-950-5975

Fax: 82-53-950-5975

Address: Department of Veterinary Pathology, College of Veterinary Medicine, Kyungpook National University, Daegu 41566

The authors declare no conflict of interest

**Supplementary figure and supplementary figure legend**

**Figure S1.** **FACS analysis of iMSCs.** The iMSCs showed high expression of α7-integrin. Also iMSCs showed two peaks in mesenchymal stem cell markers including Sca1, CD73 and CD106. Data are shown as the mean ± S.E.M.

**Figure S2. Osteogenic differentiation of iMSCs.** (A) Alizarin red staining of MEF, iMSCs and sort- iMSCs. The cells were incubated in osteogenic differentiation media for 21 days and stained with Alizarin red. The iMSCs showed osteogenic differentiation but, the sort-iMSCs. (B) Relative mRNA expression of Alkaline phosphatase (ALP) and Osterix of MEF and iMSCs.

**Figure S3. WST-1 proliferation assay of iMSCs and MDSC.** (A) Image of WST-1 proliferation assay. (B) The data of WST-1 proliferation assay measured at 450nm.

**Supplementary Tables Legends**

**Table S1. Lists of differentially expressed genes (DEGs).** The DEGs identified from the following three comparisons were shown: MDSC versus MEF, iMSC versus MEF, and sort-iMSC versus MEF. For each DEG, Entrez ID, gene symbol and description were shown together with the cluster (C1 to C23) and the group (G1 to 6) to which the gene belongs, as well as log_2_-fold-changes and P-values from the three comparisons.

See attached excel file named “Supplementary Table S1.xlsx.”

**Table S2. Clusters of DEGs identified from the three comparisons.** Twenty three clusters of DEGs (C1-C23) were identified based on up- and down-regulated genes in the three comparisons.

See attached excel file named “Supplementary Table S2.xlsx.”

**Table S3. Gene ontology biological process (GOBPs) enriched by G1-6 and the genes up- and down-regulated in MDSCs, compared to MEFs.** For each GOBP, the description and the enrichment P-value (P-value) were shown together with the number (Gene_Count) and the list (Gene) of DEGs annotated with the GOBP. The GOBPs enriched by G1-6 (P-value ≤0.05) are highlighted in magenta (GOBP_enrich).

See attached excel file named “Supplementary Table S3.xlsx.”
