# Supplementary figures and images for: Establishment of stably expandable induced myogenic stem cells by four transcription factors
Source: Cell Death Dis. 2018 Oct 25;9(11):1092. doi: 10.1038/s41419-018-1114-8 (PMC6202407; doi:10.1038/s41419-018-1114-8)

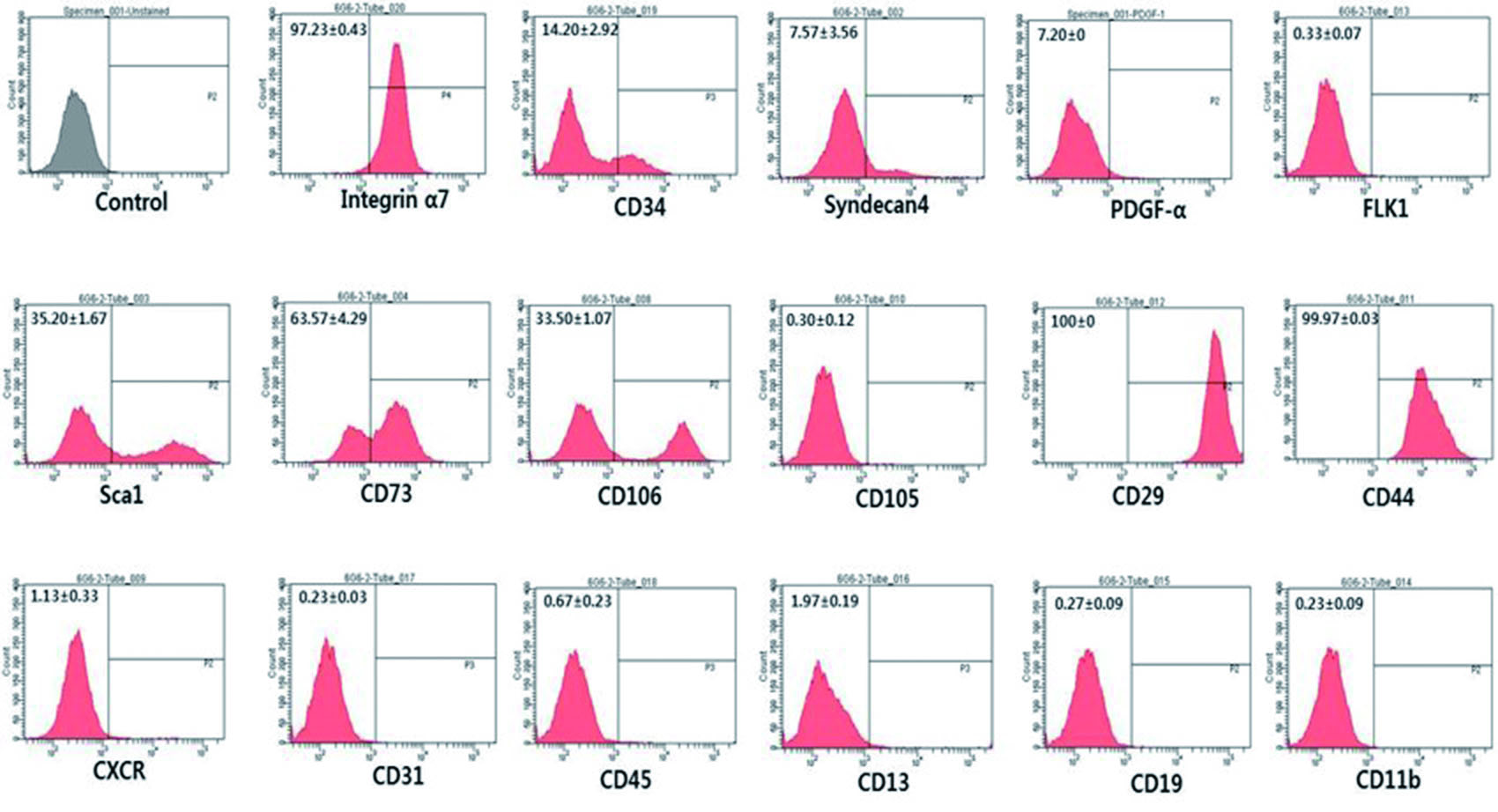

Supplement: Supplementary file 2 — Supplementary Figure S1 [file 41419_2018_1114_MOESM2_ESM.jpg]

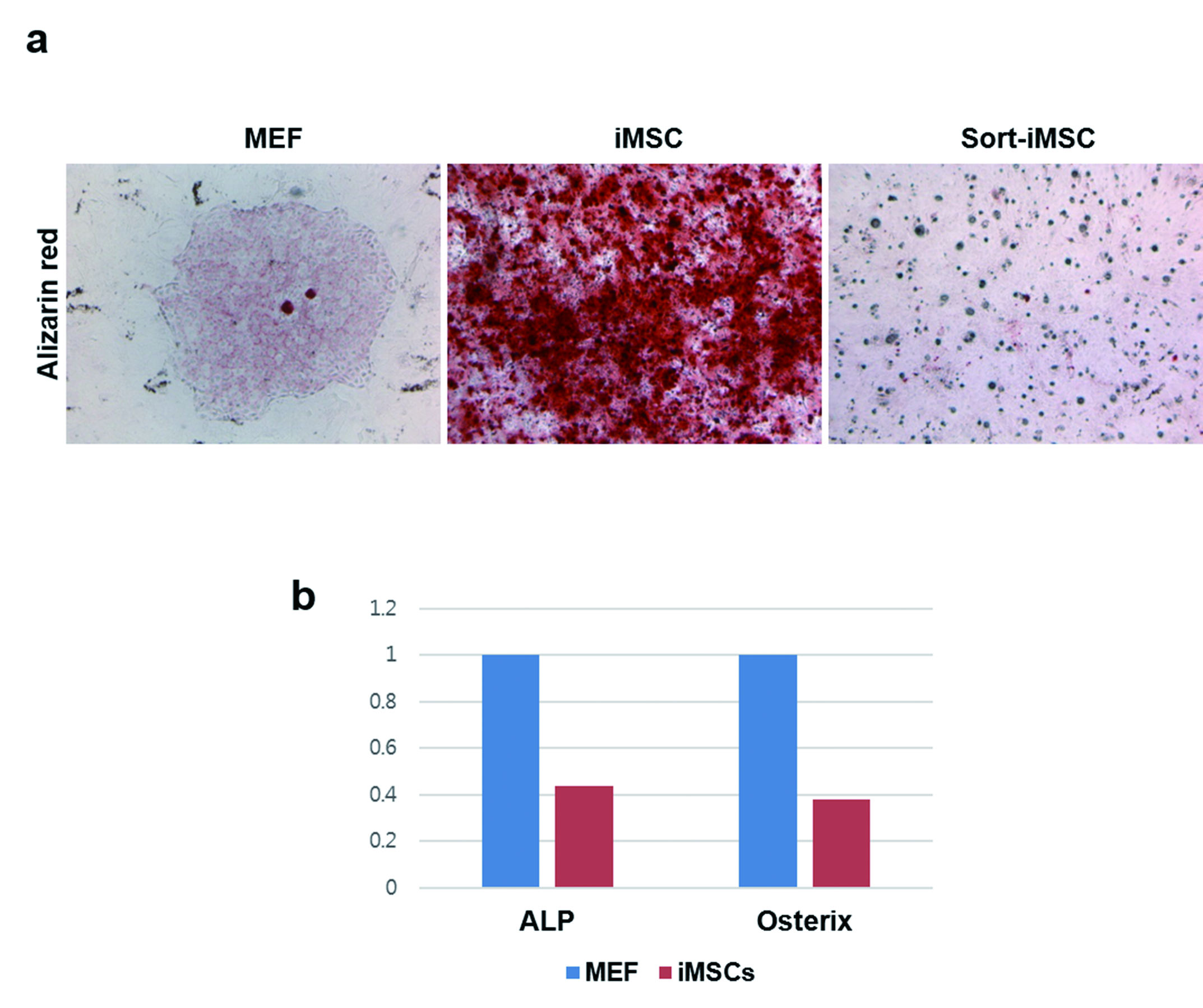

Supplement: Supplementary file 3 — Supplementary Figure S2 [file 41419_2018_1114_MOESM3_ESM.tif]

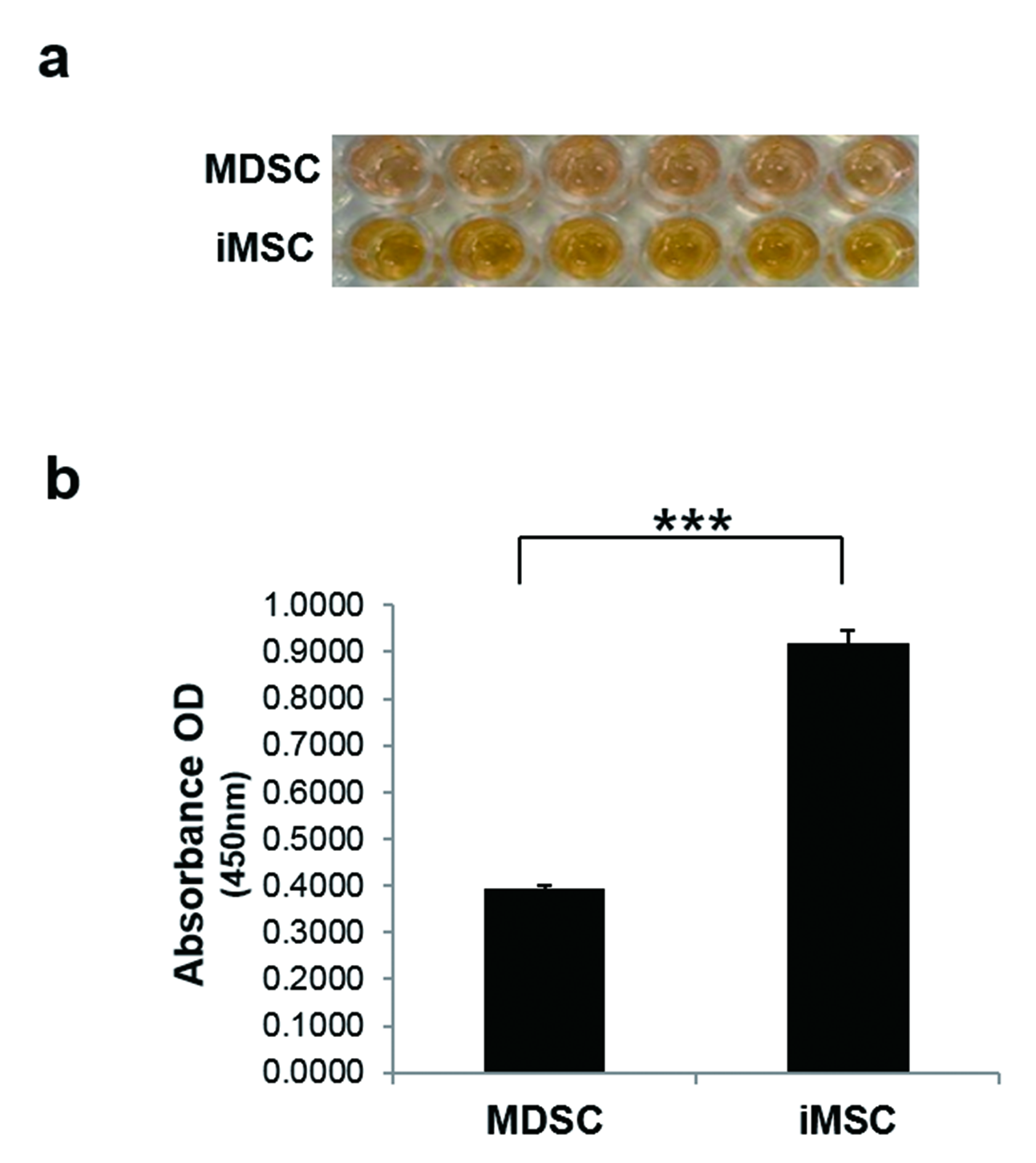

Supplement: Supplementary file 4 — Supplementary Figure S3 [file 41419_2018_1114_MOESM4_ESM.tif]
